# Study of Catalytic Combustion of Chlorobenzene and Temperature Programmed Reactions over CrCeOx/AlFe Pillared Clay Catalysts

**DOI:** 10.3390/ma12050728

**Published:** 2019-03-02

**Authors:** Yingnan Qiu, Na Ye, Danna Situ, Shufeng Zuo, Xianqin Wang

**Affiliations:** 1 Zhejiang Key Laboratory of Alternative Technologies for Fine Chemicals Process, Shaoxing University, Shaoxing 312000, China; jadeqyn@163.com (Y.Q.); yena@usx.edu.cn (N.Y.); situdn@163.com (D.S.); 2 Department of Chemical, Biological and Pharmaceutical Engineering, New Jersey Institute of Technology, Newark, NJ 07102, USA

**Keywords:** AlFe-pillared clay, CrCeOx, chlorobenzene, catalytic combustion, temperature-programmed reaction

## Abstract

In this study, both AlFe composite pillaring agents and AlFe pillared clays (AlFe-PILC) were synthesized via a facile process developed by our group, after which mixed Cr and Ce precursors were impregnated on AlFe-PILC. Catalytic combustion of organic pollutant chlorobenzene (CB) on CrCe/AlFe-PILC catalysts were systematically studied. AlFe-PILC displayed very high thermal stability and large BET surface area (*S*_BET_). After 4 h of calcination at 550 °C, the basal spacing (*d*_001_) and *S*_BET_ of AlFe-PILC was still maintained at 1.91 nm and 318 m^2^/g, respectively. Large *S*_BET_ and *d*_001_-value, along with the strong interaction between the carrier and active components, improved the adsorption/desorption of CB and O_2_. When the desorption temperatures of CB and O_2_ got closer to the CB combustion temperature, the CB conversion could be increased to a higher level. CB combustion on CrCe/AlFe-PILC catalyst was determined using a Langmuir–Hinshelwood mechanism. Adsorption/desorption/oxidation properties were critical to design highly efficient catalysts for CB degradation. Besides, CrCe/AlFe-PILC also displayed good durability for CB combustion, whether in a humid environment or in the presence of volatile organic compound (VOC), making the catalyst an excellent material for eliminating chlorinated VOCs.

## 1. Introduction

Chlorinated volatile organic compounds (CVOCs) are considered to be very harmful to the environment, not only a direct harm on human health but also destroy the ozone layer [1,2]. Today, the major industrial processes for CVOCs elimination involve direct combustion at very high temperatures (above 850 °C). This is a fairly expensive process and produces highly toxic byproducts or intermediates by incomplete combustion, such as dioxins, Cl_2_, and CO [2,3]. The low operating temperatures (<500 °C) and high selectivity into harmless product, make catalytic combustion an attractive option [4,5,6].

Due to the high toxicity of dioxins and the need for laboratory safety, model reagents, such as chlorobenzene (CB), are used to predict destruction behavior of dioxins on different catalysts [7,8]. Vanadia-based catalysts [9,10], precious metals (Pt, Pd, Ru) supported on zeolites [11,12], and various oxides [13,14] are employed for the catalytic combustion process. However, these catalysts often have some disadvantages of relative low catalytic performance, rapid deactivation caused by coking or chlorine poisoning, high price, and the formation of polychlorinated benzene [14]. Transition metal oxide catalysts including cobalt, copper, manganese, and chromium oxides can not only resist deactivation caused by chlorine poisoning but also enhance catalytic activities by the modification with rare earth elements [15,16]. Doping ceria into transition metals oxides could improve redox property of metal oxides, increase the mobility of oxygen and the rate of chlorine removal or transfer, thereby enhancing their catalytic properties and reaction stability during CVOCs combustion [17]. The catalytic behavior is associated with the interaction between the catalyst and the reactant, thus, understanding the relationship between adsorption/desorption and combustion of CVOCs over the catalysts becomes very important. The information can not only explain their own catalytic characteristics, but also provide insights into catalytic macroscopic behaviors [18,19,20].

Catalytic combustion is a typical gas–solid, two-phase reaction, which mostly occurs on the surface of catalysts. The advantage of heterogeneous catalysis is that the catalyst does not need to be separated, so the process can be operated in uninterrupted flow. Nevertheless, if there is a need to separate the catalyst, it can be done in a much simpler way than a homogeneous catalyst. Heterogeneous catalysts have been well applied in many fields such as heterogeneous Suzuki cross-coupling reaction catalyzed by magnetically recyclable nanocatalyst [21], modulated large-pore mesoporous silica as an efficient base catalyst for a Henry reaction [22], and magnetically separable and sustainable nanostructured catalysts for heterogeneous reduction of nitroaromatics [23,24,25]. The disadvantage of heterogeneous catalysis is that the catalyst can only use the catalytic active points on its surface, which is slightly inefficient, but can be improved by increasing the specific surface area (*S*_BET_). Therefore, the active components are usually dispersed on the carrier with large *S*_BET_. In a catalytic combustion reaction, the carrier can not only support the dispersed active components, but also increase the stability, selectivity, and activity of catalysts. The support can also reduce the use of high-priced active components, thereby reducing the cost of the catalyst. Common metal oxide catalyst carriers include γ-Al_2_O_3_, TiO_2_, SiO_2_, ZrO_2_, or their complexes. Molecular sieve supported catalysts also show good catalytic combustion activity. The molecular sieve carriers studied include ZSM-5, β-molecular sieve, SBA-15, MCM-41, and so on.

Natural clay is a hydrated aluminosilicate, which can be designed by crosslinking or pillaring, which leads to the formation of a class of material known as pillared clays (PILCs). Moreover, because of its wide distribution, abundant reserves, and low price, it has its own advantages in replacing existing catalyst carriers. PILCs have large *S*_BET_, uniform pore size distribution, and high thermal stability. They are good catalytic materials and carriers. A popular research topic in recent years is synthesizing PILCs supported catalysts for VOCs catalytic combustion reactions [26,27,28], whereas the application for CVOCs combustion is rare and the structure-activity relationship was not clear. It has been reported in the literature that *S*_BET_, pore volume (*V*_p_), and thermal stability of PILC can be improved by the synthesis composite pillaring agents, and these mixed pillaring agents has been widely used in the past 30 years [29]. At present, many kinds of mixed pillaring agents have been synthesized, with aluminum pillaring agents being one of the components. However, defects in the preparation process still exist. Therefore, there is an urgent need to optimize involved unit operations and simplify procedures, especially to reduce the amounts of NaOH and AlCl_3_ solutions.

The present work intends to simplify the synthesis steps, using a high-temperature hydrothermal one-step method, to prepare AlFe composite pillaring agents, and then synthesize AlFe-PILC [30]. Compared with Na-Mt, AlFe-PILC had good structural characteristics, such as larger *S*_BET_ and *V*_p_, so it had the potential for use as a catalytic support. How to improve the efficiency of low temperature catalytic combustion of CB and improve the stability of the catalysts are the key problems to be solved in the current catalytic combustion process. This paper intends to use AlFe-PILC as a carrier to prepare CrCeOx catalysts, and to study their application in the catalytic combustion reaction of CB, aiming at forming achievements in advanced catalytic materials and their applications. The adsorption/desorption properties and catalytic performance of CB were systematically studied in order to get a clear map about the structure–activity relationship for the catalytic reaction and explore its potential for further industrial application.

## 2. Experimental Section

### 2.1. AlFe-PILC and Catalyst Preparation

The starting material was the sodium form of montmorillonite (Na-Mt) (>100 mesh, Hengsheng Trading Co., Ltd., Baotou, China). AlFe pillaring agents were prepared with Locron L (Clariant, Switzerland, Al_2_(OH)_5_Cl·2-3H_2_O) and ferric nitrate solution. The following preparation of AlFe pillaring (the molar ratio of Al/Fe is 5) and AlFe-PILC using the similar method detailed in our previous research [30].

Cr/Na-Mt, CrCe(5:1)/Na-Mt, Ce/AlFe-PILC, Cr/AlFe-PILC, and CrCe/AlFe-PILC were synthesized by impregnating a Cr and Ce nitrate solution onto the equal volumes of supports (Na-Mt and AlFe-PILC) overnight, followed sequentially by drying and calcination at 500 °C for 2 h. Cr, Ce, or CrCe loading for each catalyst was 10 wt.%, and Cr/Ce molar ratios were adjusted to 2.5:1, 5:1, 7.5:1, and 10:1, respectively. All the reagents were analytically pure, and obtained by Shanghai Chemical Reagent Factory (Shanghai, China).

### 2.2. Characterization

The samples were characterized using X-ray diffraction (XRD) (PANalytical, Almelo, Netherlands) for *d*_001_ value and phase composition. The *S*_BET_, mesopore area (*A*_mes_), *V*_p_, micropore volume (*V*_mic_), and pore size distribution of the samples were determined via N_2_ adsorption isotherms using a TristarII 3020 apparatus (Micromeritics Company, Atlanta, GA, USA). High-resolution transmission electron microscopy (HRTEM) on a JEM-2100F (JEOL, Valley, Japan) was employed to get the catalyst morphology and particle size. The chemical compositions of the catalysts were determined with energy dispersive X-ray spectroscopy (EDS) using an Oxford INCA instrument (Oxford Instruments, Warrington, UK). All the characterization methods for the samples have been reported and detailed in our previous research [30,31,32].

### 2.3. Catalytic Performance Tests and Temperature-Programmed Reactions

The activity of the catalysts was evaluated in a WFS-3010 microreactor (Xianquan, Tianjin, China). The degradation products were detected by mass spectrometry (MS, QGA, Hiden, U.K.). No byproduct other than H_2_O, CO_2_, and HCl was detected. Thus, the conversion was calculated based on CB consumption [31]. To further study the “mixture effect” of the feed gases, 1% (*v*/*v*) water vapor and 100 ppm toluene were also introduced. Besides, the durability of CrCe (5:1)/AlFe-PILC for the catalytic combustion of CB was investigated at a CB concentration of 500 ppm and gas hourly space velocity (GHSV) of 25,000 h^−1^.

H_2_ temperature-programmed reduction (H_2_-TPR) was conducted on a CHEMBET-3000 instrument (Quantachrome, Boynton Beach, FL, USA) to evaluate the reducibility of the catalysts. The sample (50 mg) was pre-treated in air at 300 °C for 0.5 h, and then the temperature was reduced to 100 °C. The flow rate of the reductive gas (5 vol.% H_2_/Ar, purified by deoxidizer and silica gel) was 40 mL/min, and the reaction temperature was elevated by 7.5 °C/min. The H_2_ uptake was determined using a thermal conductivity detector (TCD) detector (Shimadzu, GC-14C, Kyoto, Japan), and the H_2_O produced was absorbed using 5 Å zeolite [32].

Temperature-programmed desorption (TPD) and temperature-programmed surface reaction (TPSR) measurement were carried out in the same equipment as the catalytic performance tests to determine the adsorption capacity and the relationship between desorption performance and catalytic combustion properties [30,32]. Prior to the measurement, 350 mg catalyst was pre-treated in Ar (99.99%) at 300 °C for 30 min, then the temperature was decreased to 50 °C. Adsorption gas (40 mL/min) was a mixture of Ar (99.99%) and CB (about 500 ppm). The quantitative amounts were estimated by integrating the desorption curve. After the adsorption reached an equilibrium (CB concentration in the effluent gas was monitored using Gas Chromatography-Mass Spectrometry (GC-MS), the catalysts were purged by Ar (99.99%) for a period of time at 50 °C until CB concentration to constant. Then, the desorption and catalytic properties of the catalysts were measured from 50 to 500 °C with a heating rate of 7.5 °C/ min in 20 vol.%O_2_/80 vol.%Ar (without CB). The reactants and products (such as CB (m/z =112), CO_2_ (44), H_2_O (18), Cl_2_ (71), and HCl (36.5) were analyzed with an on-line MS apparatus.

O_2_ temperature-programmed desorption (O_2_-TPD) was also performed using the same apparatus. The catalyst (350 mg) was firstly treated in 10 vol.%O_2_/90 vol.%Ar at 300 °C for 0.5 h. After the temperature was slowly cooled down to room temperature, followed by an Ar purge (40 mL/min) for 30 min, the sample was heated from 50 to 900 °C with a heating rate of 7.5 °C/min in Ar flow. The signal of desorbed oxygen was monitored by the MS.

## 3. Results and Discussion

### 3.1. Material Textural Properties

#### 3.1.1. XRD Analysis

**Figure 1:**
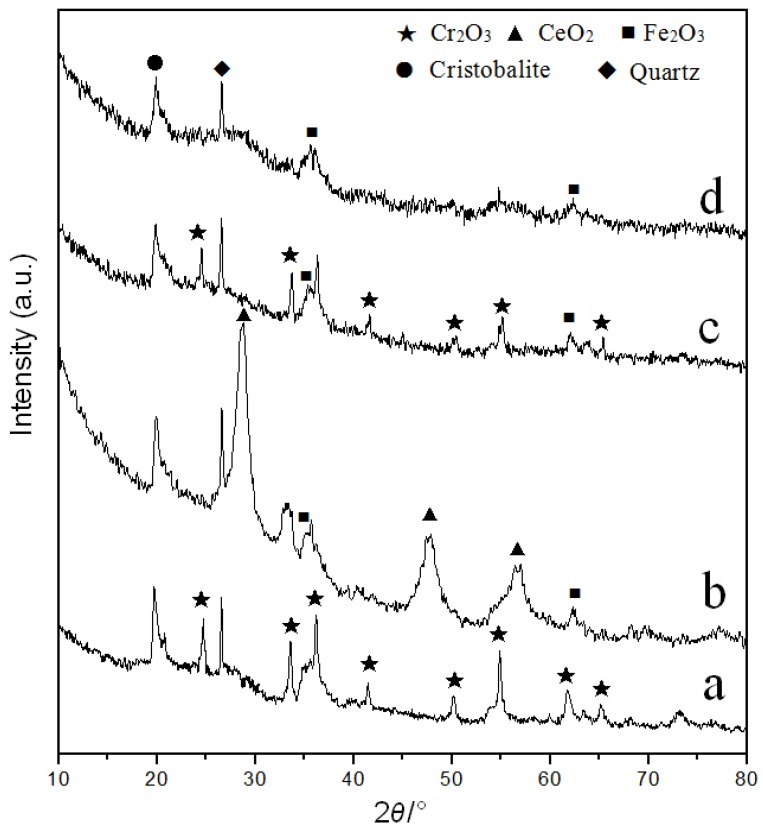
XRD patterns of the catalysts: (**a**) Cr/Na-Mt, (**b**) Ce/AlFe-PILC, (**c**) Cr/AlFe-PILC, and (**d**) CrCe(5:1)/AlFe-PILC.

Figure 1 presented the XRD patterns (2*θ*: 10–80°) of the Cr/Ce catalysts supported by Na-Mt and AlFe-PILC. The diffraction peaks belonging to cristobalite and quartz appear at 19.8° and 26.7° (2*θ*), respectively [33]. Cristobalite and quartz are two of the main components of montmorillonite. They have the characteristics of high temperature resistance, which can ensure the stability of the catalysts in a high temperature gas–solid continuous reaction. Therefore, they play an important role in catalyst components. The diffraction peaks of Fe_2_O_3_ appeared in all the AlFe-PILC based catalysts because the amount of Fe_2_O_3_ increased after AlFe pillaring process. The diffraction peaks of CeO_2_ appeared in Ce/AlFe-PILC catalyst. Compared with Cr/Na-Mt, the diffraction peak intensity of Cr_2_O_3_ in Cr/AlFe-PILC clearly decreased, and the result showed that the dispersion of Cr_2_O_3_ particles on AlFe-PILC was greatly improved. After adding Ce, the diffraction intensity of Cr_2_O_3_ for CrCe(5:1)/AlFe-PILC further decreased. On the one hand, the addition of Ce was beneficial to the dispersion of Cr_2_O_3_, and on the other hand, it may have been due to the reduction of Cr_2_O_3_ content. Notably, the CeO_2_ diffraction peaks were not found in CrCe(5:1)/AlFe-PILC, possibly because the small amount of CeO_2_ was highly dispersed on AlFe-PILC.

#### 3.1.2. N_2_ Adsorption/Desorption

Table 1 summarizes the textural properties of samples. *S_BET_* and *A*_mes_ of Na-Mt were only 51 and 41 m^2^/g, and the values of *V*_p_ and *V_mic_* were 0.076 and 0.0043 cm^3^/g, respectively. AlFe-PILC’s *S_BET_* and *V*_p_ reached 318 m^2^/g and 0.195 cm^3^/g, respectively, indicating that the formed composite AlFe polycation was relatively large and thus the clay layers were further stripped to form more porous structures. The *V_mic_* of AlFe-PILC was 0.077 cm^3^/g, and it was about 39.5% in *V*_p_. Compared with the Na-Mt and AlFe-PILC support, the supported Cr or CrCe catalysts exhibited lower *S_BET_* and *V*_p_ values, indicating that some of the Cr and Ce ions migrated into the pores and clay layers, and thus blocked some of the pores. It was worth noting that a large number of micro-mesoporosity in AlFe-PILC support was favorable for good dispersion of active species and rapid diffusion of reactants, thus significantly enhanced their catalytic activity of various reactions.

**Figure 2:**
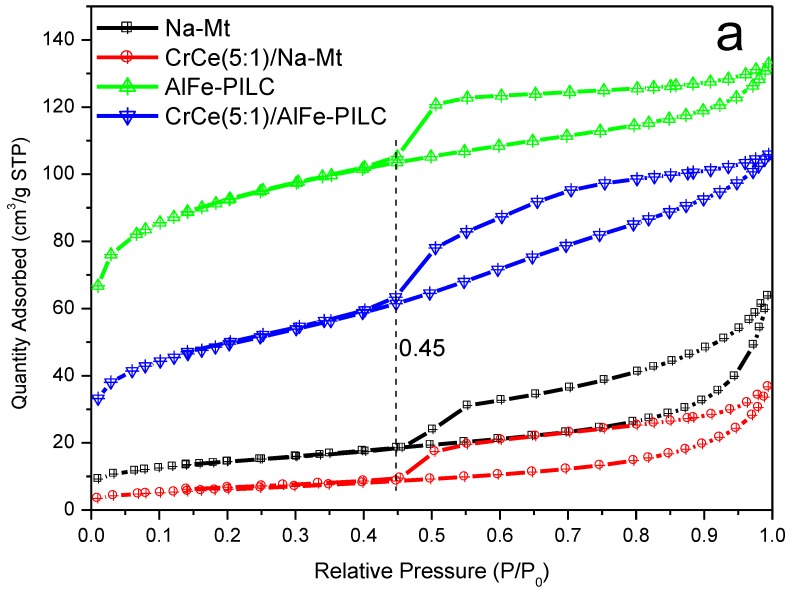

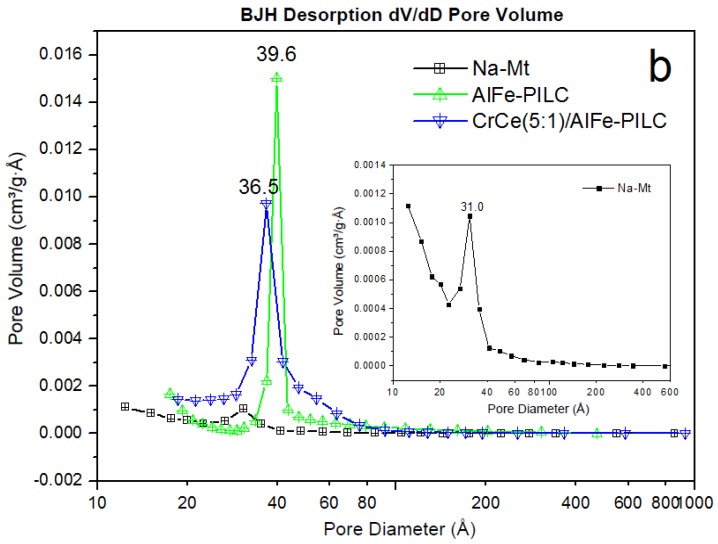
Characteristics of the samples: (**a**) N_2_ adsorption/desorption isotherms, and (**b**) pore-size distributions.

In Figure 2a, N_2_ adsorption/desorption isotherms for all the materials were type IV, while its type H3 adsorption–desorption hysteresis appeared at P/P_0_ above 0.45, indicating that the material had a mesoporous structure and the pores in the material were slit pores formed by layer-like structures. The adsorption amount of Na-Mt was low; however, AlFe-PILC had a pronounced increase in adsorption because more pores were formed by AlFe polyoxycations. The addition of Cr_2_O_3_ and CeO_2_ to Na-Mt and AlFe-PILC decreased N_2_ adsorption capacity and thus pore volume, indicating the doped cations entered and/or blocked the pores of Na-Mt and AlFe-PILC. In Figure 2b, the average mesoporous diameters of AlFe-PILC materials were distributed in a narrow range of 3.96 nm and were wider than the pore-diameter distribution range of Na-Mt (3.10 nm), confirming the pore size was increased after pillaring. The average pore size of CrCe(5:1)/AlFe-PILC was in a narrow region of approximately 3.65 nm. The stability of AlFe-PILC support was good and the mesoporous structure was not destroyed.

#### 3.1.3. HRTEM Analysis

**Figure 3:**
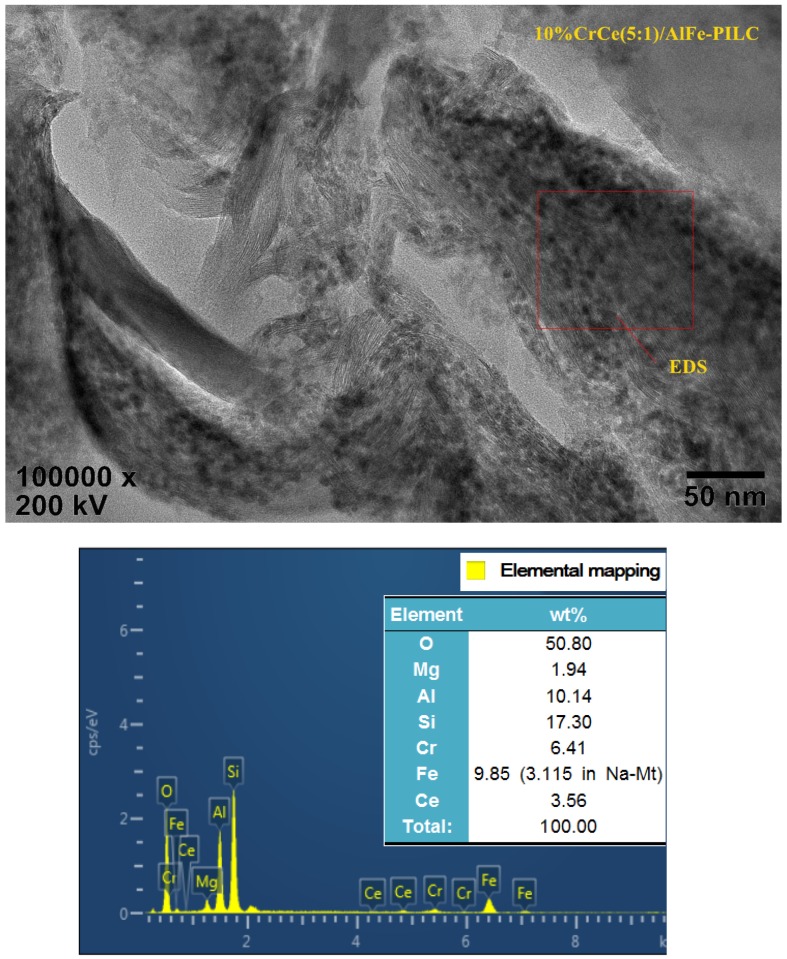
HRTEM picture and the EDS spectra of CrCe(5:1)/AlFe-PILC.

Figure 3 shows HRTEM picture and the EDS spectra of CrCe(5:1)/AlFe-PILC. It can be seen that CrCe(5:1)/AlFe-PILC had a layered structure and the active particles (5–10 nm in size) were uniformly distributed throughout the support, and the layered structure of AlFe-PILC was not damaged after loading active ingredients. Al, Fe, Cr, Ce, O, and other elements were identified in the EDS spectra, which confirmed that the active species (Cr and Ce) were successfully loaded on the surface of AlFe-PILC. The results indicated that AlFe-PILC was a good support for highly dispersed active species. All these properties were conducive to improving the catalytic degradation of CB.

### 3.2. Catalytic Performance Test

#### 3.2.1. CB Combustion and Durability Test

**Figure 4:**
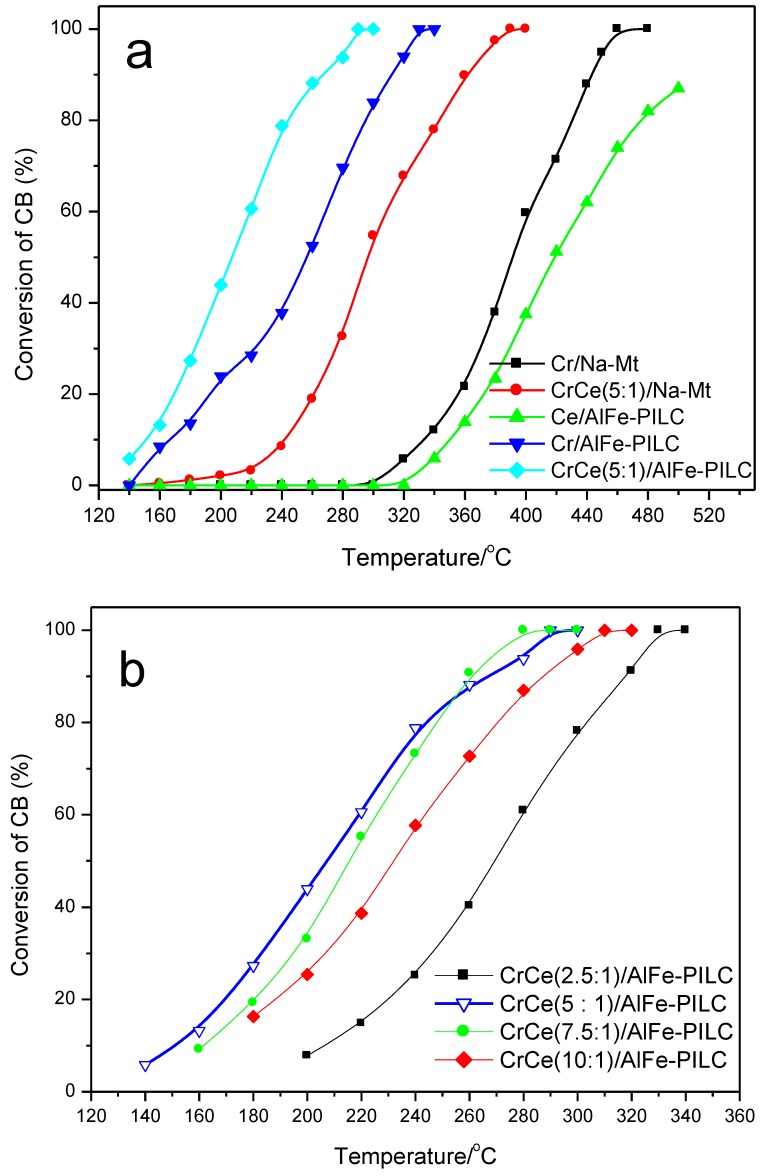
(**a**) CB conversions vs. temperature over Cr/Na-Mt, CrCe(5:1)/Na-Mt, Ce/AlFe-PILC, Cr/AlFe-PILC, and CrCe(5:1)/AlFe-PILC. (**b**) The effect of Cr/Ce molar ratios on CB catalytic combustion over CrCe/AlFe-PILC.

Figure 4 presents the conversions of CB combustion on various catalysts. In Figure 4a, Cr/Na-Mt exhibited poor performance and did not fully convert CB until 460 °C. Cr/AlFe-PILC caused complete degradation of CB at 320 °C, about 140 °C lower than the degradation temperature of CB required for Cr/Na-Mt. The conversion of Ce/AlFe-PILC was negligibly low, and it was 87%, even at a reaction temperature of 500 °C. Ceria doping significantly improved the catalytic activities of Cr/Na-Mt and Cr/AlFe-PILC. In addition, the molar ratios of Cr/Ce (2.5, 5, 7.5, and 10) had an effect on the catalytic performance of CrCe/AlFe-PILC (Figure 4b). The catalysts exhibited a lower performance when the Cr/Ce ratio was less than 5, possibly indicating Cr_2_O_3_ was the active species and CeO_2_ acted as an assistant. The catalyst performance decreased when the Cr/Ce molar ratio was larger than 5, possibly because less oxygen vacancies existed with a relatively lower amount of CeO_2_. Therefore, the content of CeO_2_ was one of the key factors to improving the performance of CrCe/AlFe-PILC. In particular, CrCe(5:1)/AlFe-PILC had the highest catalytic performance and could completely degrade CB at about 290 °C. No Cl_2_ or other byproducts were detected, showing that the catalyst had good selectivity for HCl without producing secondary pollution.

**Figure 5:**
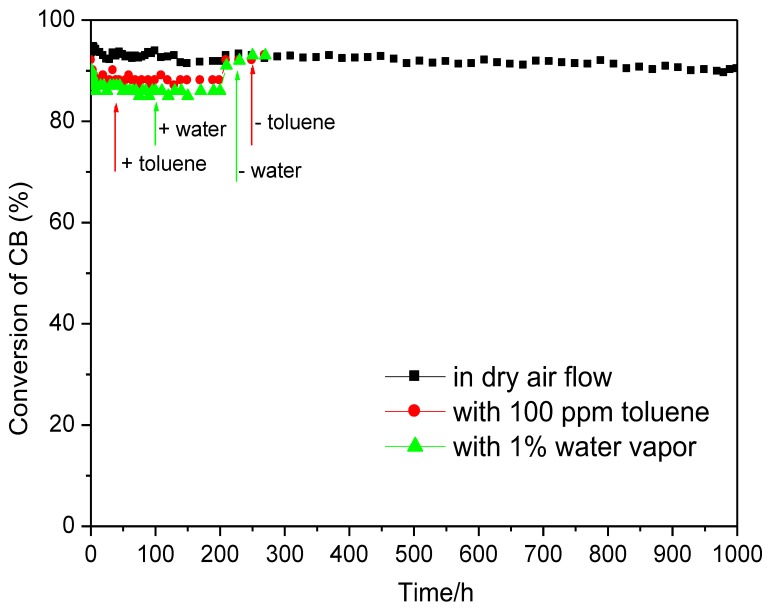
Lifetime test performed for CrCe(5:1)/AlFe-PILC at 280 °C. CB concentration: 500 ppm; gas hourly space velocity (GHSV): 25,000 h^−1^; catalyst amount: 350 mg.

Figure 5 shows the curves of CB over CrCe(5:1)/AlFe-PILC in the continuous reaction process. There was no significant drop for catalytic activities within 1000 h tests, suggesting that the CrCe(5:1)/AlFe-PILC catalyst was durable. Moreover, this catalyst also displayed good catalytic performances in the presence of 100 ppm toluene or 1% water vapor, further indicating its high potential for industrial application.

#### 3.2.2. Effect of CB Concentration and Gas Hourly Space Velocity

**Figure 6:**
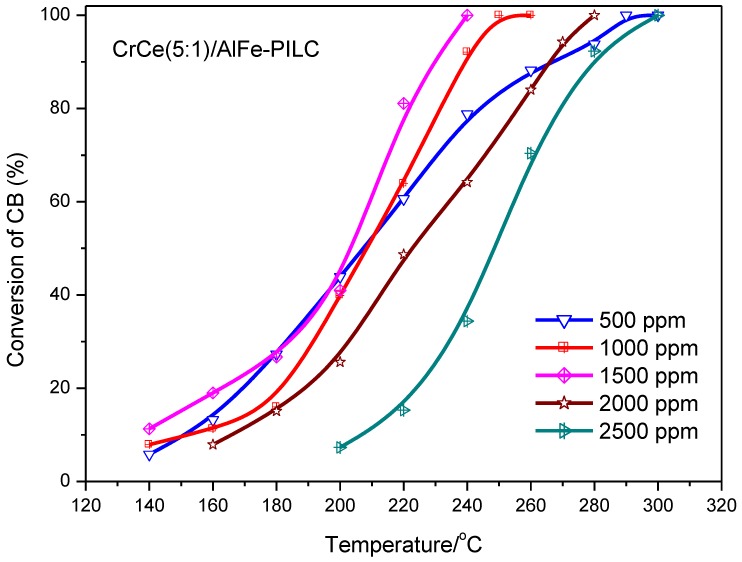
The effect of inlet concentration on CB catalytic combustion over CrCe(5:1)/AlFe-PILC. CB concentration: 500–2500 ppm; GHSV: 25,000 h^−1^; catalyst amount: 350 mg.

Figure 6 presents the effect of CB inlet concentrations on the catalytic performance of CrCe(5:1)/AlFe-PILC. The change of its inlet concentration had a great influence on CB conversion from 500 to 2500 ppm. Furthermore, when the inlet concentration was in the range of 500 to 1500 ppm, CB conversion increased appreciably. This was primarily because low concentration CB only provided a small amount for chemisorbed CB on catalyst active sites and could act as the controlling factor of the reaction. However, as the concentration of CB continued to increase, CB conversion decreased until it was completely prohibited, which may be related to chemisorbed oxygen on the catalyst active sites becoming the reaction controlling factor [34]. The result indicated that CB degradation combustion proceeds via a Langmuir–Hinshelwood (L-H) mechanism, and this catalyst could be used for removing CB waste gases with a wide range of concentrations.

**Figure 7:**
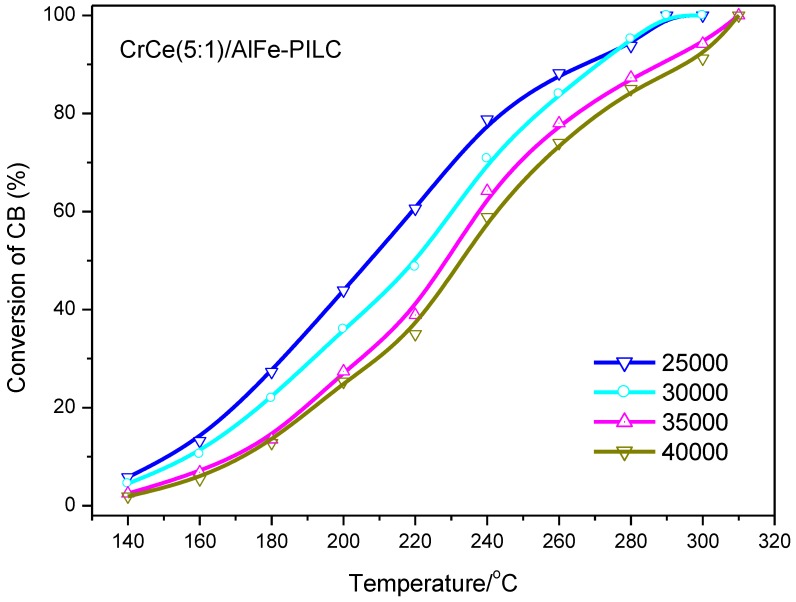
CB conversions vs. temperature over CrCe(5:1)/AlFe-PILC under the conditions of CB concentration at 500 ppm and GHSV at 25,000–40,000 h^−1^.

Figure 7 shows the effect of GHSV on the CB catalytic combustion activities over CrCe(5:1)/AlFe-PILC. GHSV is the gas hourly space velocity. To calculate this parameter, the flow rate of feed gas (involved inert and main components) can be adjusted. Then, GHSV is the ratio of gas flow rate in standard conditions to the volume of the catalyst. Increasing GHSV slightly decreased the catalytic performance, indicating that this catalyst was highly effective for CB destruction in different reaction conditions. The catalyst active sites were already fully occupied, even with the lowest GHSV used in this work, and more reactant molecules provided by high GHSV could not be chemisorbed and reacted. Thus, high temperature was required to obtain the same conversion with high GHSV.

### 3.3. Temperature-Programmed Reaction Studies

#### 3.3.1. H_2_-TPR Analysis

**Figure 8:**
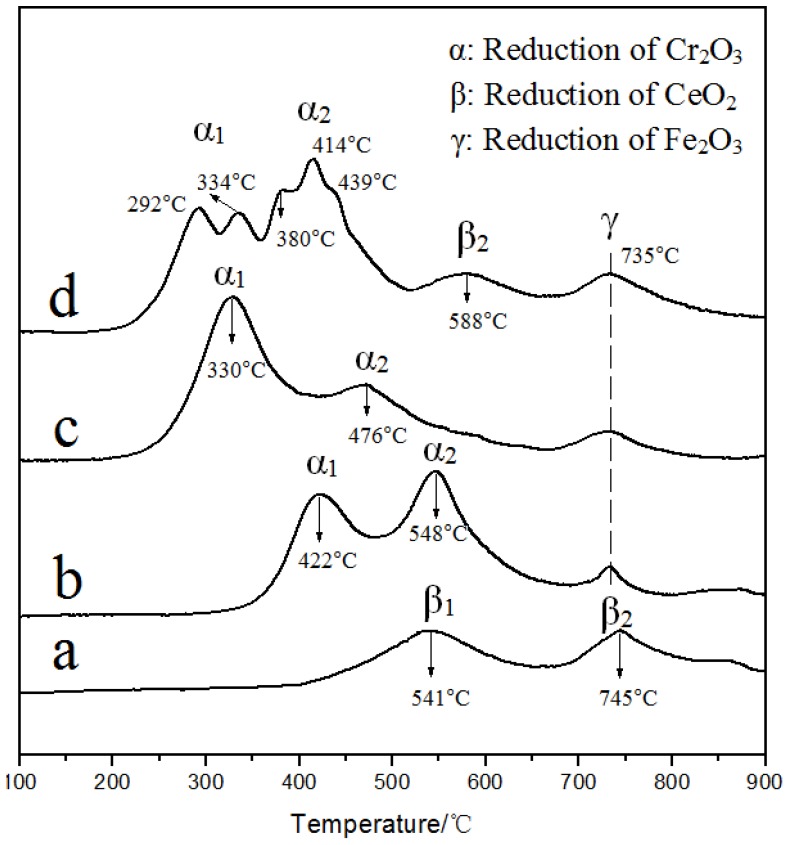
H_2_-TPR spectra: (**a**) Ce/Na-Mt, (**b**) Cr/Na-Mt, (**c**) Cr/AlFe-PILC, and (**d**) CrCe(5:1)/AlFe-PILC.

H_2_-TPR profiles of the catalysts are shown in Figure 8. The reduction of Fe_2_O_3_ species was obvious in all the catalysts (γ peak), which was from the relatively high contents of Fe_2_O_3_ (4.45% in the original clay) [35,36]. Compared with the γ peak area from Cr/Na-Mt, the areas from Cr/AlFe-PILC and CrCe(5:1)/AlFe-PILC increased, revealing that more iron oxide species were formed as Fe_2_O_3_ pillars in AlFe pillaring. In the case of Ce/Na-Mt, it was beneficial for the reduction of surface and bulk CeO_2_ to have two reduction peaks at 541 and 745 °C. There were two reduction peaks below 650 °C in the Na-Mt and AlFe-PILC-supported Cr catalysts, which indicated that peaks α_1_ and α_2_ were the reduction peaks of the surface and inside Cr_2_O_3_, respectively. For CrCe(5:1)/AlFe-PILC, peaks α_1_ and α_2_ were divided into two or three peaks, which suggested the better-dispersed Cr_2_O_3_ on the AlFe-PILC support. Compared with Cr/Na-Mt, the reduction peaks of CrCe(5:1)/AlFe-PILC systematically shifted to lower temperatures, indicating CeO_2_ improved the reducibility of Cr_2_O_3_ by increasing Cr_2_O_3_ dispersion and lattice oxygen mobility. The peak β_2_ of CrCe(5:1)/AlFe-PILC at 588 °C was the reduction peak of bulk CeO_2_, and the peak of surface CeO_2_ overlapped with peak α_2_. The CeO_2_ reduction peak was shifted toward lower temperatures compared with that from Ce/Na-Mt. This shift occurred because Cr_2_O_3_ underwent a stronger oxidation process and could be more easily reduced, allowing it to interact with CeO_2_ to produce a reduction peak at a lower temperature. It suggested that the interaction between Cr_2_O_3_ and CeO_2_ species weakened the Ce-O bond and promoted the reduction of CeO_2._ The α peak temperatures followed: Cr/Na-Mt > Cr/AlFe-PILC > CrCe(5:1)/AlFe-PILC. The results indicated that the interaction between Cr_2_O_3_ and CeO_2_ species could improve the mobility of oxygen species in the catalysts, thus improving the reduction of both Cr_2_O_3_ and CeO_2_ species.

#### 3.3.2. TPD and TPSR Analysis

**Figure 9:**
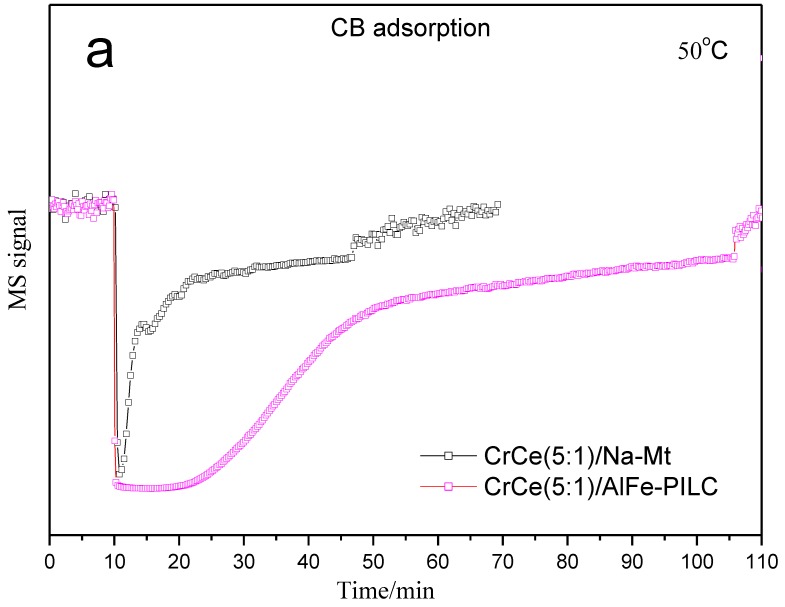

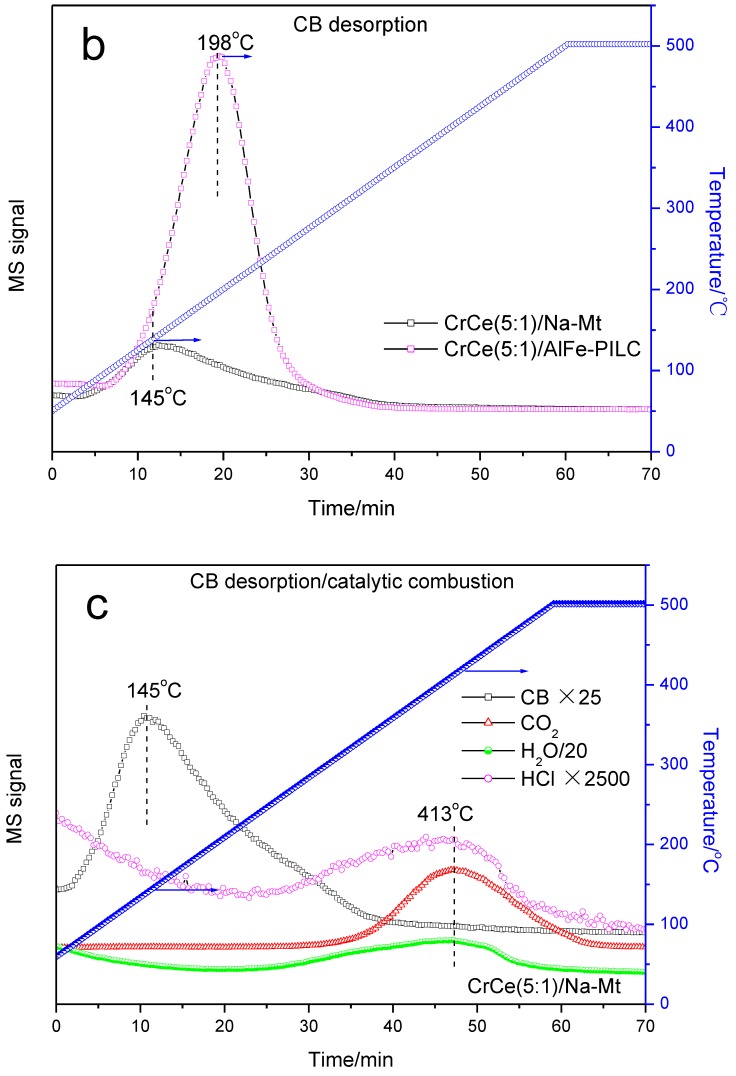

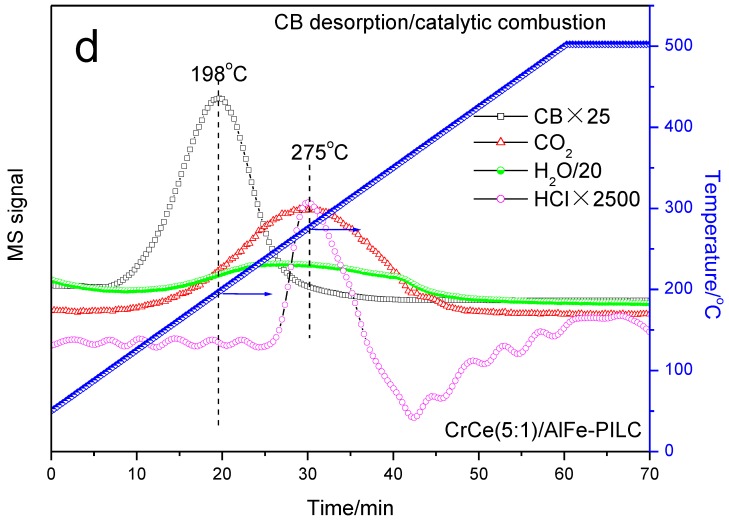
Performance of CB adsorption/desorption and catalytic combustion over CrCe(5:1)/Na-Mt and CrCe(5:1)/AlFe-PILC. (**a**) CB adsorption of CrCe(5:1)/Na-Mt and CrCe(5:1)/AlFe-PILC; (**b**) CB desorption of CrCe(5:1)/Na-Mt and CrCe(5:1)/AlFe-PILC; (**c**) CB desorption/catalytic combustion of CrCe(5:1)/Na-Mt; (**d**) CB desorption/catalytic combustion of CrCe(5:1)/AlFe-PILC.

The adsorption/desorption of CB, catalytic combustion behavior, and the evolution of the main products (CO_2_, H_2_O, and HCl) over the catalysts were investigated using CB-TPD/TPSR techniques (Figure 9). As it was mentioned previously, the CB combustion on CrCe(5:1)/AlFe-PILC catalyst proceeded via an L-H mechanism, where the adsorption of reactants on the catalyst active sites was a critical step. In Figure 9a, CrCe(5:1)/Na-Mt and CrCe(5:1)/AlFe-PILC showed different CB adsorption capacities. The CB absorption capacities of CrCe(5:1)/AlFe-PILC (44.8 μmol/g) was obviously stronger than CrCe(5:1)/Na-Mt (7.9 μmol/g) by integrating over the absorption spectra. The above results fully proved that clay materials with larger *S*_BET_, *V*_p_, and *d*_001_-value favor CB adsorption. In Figure 9b, the temperature of CB desorption peaked for CrCe(5:1)/Na-Mt and CrCe(5:1)/AlFe-PILC were 145 °C and 198 °C, respectively, indicating that the interaction of CB and CrCe(5:1)/AlFe-PILC was stronger than with CrCe(5:1)/Na-Mt. Therefore, CB could remain inside the pores or outside the surface of CrCe(5:1)/AlFe-PILC for a longer time, being conducive to the adsorption and catalytic degradation of CB. The results indicated that improved structure and the strong interaction between CrCe mixed oxides with AlFe-PILC enhanced the adsorption of CB.

As shown in Figure 9c,d, CB desorption was accompanied with CB combustion under O_2_/Ar, and the adsorbed CB species reacted with lattice O from CrCeO_x_ to form CO_2_, H_2_O, and HCl. CB was completely reacted over CrCe(5:1)/AlFe-PILC at about 300 °C, while it needed about 440 °C on CrCe(5:1)/Na-Mt. CO_2_, H_2_O, and HCl were detected, but CO and Cl_2_ were not detected, indicating that the catalysts in the study had high selectivity to HCl and CO_2_ formation. It was notable that the peak temperature of the products for CrCe(5:1)/Na-Mt was at 413 °C, which was much higher than that of CB desorption peak temperature (145 °C). However, the peak temperature of product for CrCe(5:1)/AlFe-PILC was at 275 °C, which was close to that of CB desorption (198 °C). This phenomenon can explain why CrCe(5:1)/AlFe-PILC had the highest CB degradation activities compared to other catalysts in this work. The larger overlapped region between CB desorption and catalytic combustion, the better the catalytic performance. Therefore, tuning the CB adsorption and catalytic properties was a key to designing an efficient catalyst for CB catalytic combustion.

**Figure 10:**
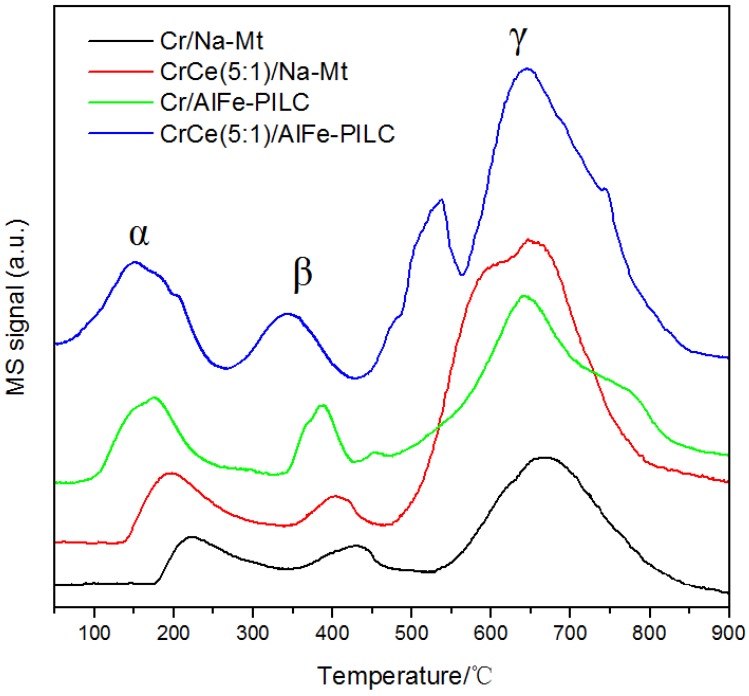
O_2_-TPD profiles of Cr/Na-Mt, CrCe(5:1)/Na-Mt, Cr/AlFe-PILC, and CrCe(5:1)/AlFe-PILC.

In order to find out the relationship between the oxygen species absorbed on the catalyst surface and the catalytic properties, O_2_-TPD were investigated from 50 to 900 °C. The O_2_-TPD plots for Cr and CrCe metal oxide catalysts consisted of oxygen desorption regions shown in Figure 10. There were three types of desorption peaks, the α desorption peak, the β desorption peak, and the γ desorption peak. Furthermore, these three peaks could be assigned to superoxide ion O_2_^−^, peroxide ion O_2_^2−^/O^−^, and lattice oxygen ion O^2−^, respectively [37,38]. Increasing the temperature is beneficial to increase the rate of desorption and transformation of superoxide species into O_2_^2−^, O^−^, and O_lattice_^2−^ [39]. It can be seen that the α and β desorption peaks follow: CrCe(5:1)/AlFe-PILC < Cr/AlFe-PILC < CrCe(5:1)/Na-Mt < Cr/Na-Mt, which was in good agreement with the aforementioned catalytic performance of CB combustion. It was worth mentioning that the total amount of surface-active oxygen species, in terms of the sum of α and β desorption areas, follows the same sequence of the peaks. It can be observed from the γ desorption peak that adding CeO_2_ increased the desorption area of lattice oxygen ion O^2−^ compared with the non-doped catalyst. Thus CrCe(5:1)/AlFe-PILC exhibited the highest oxidation performance since electrophilic O_ads_ (O_2_^−^, O_2_^2−^, O^−^) played a critical role in the complete oxidation of organic compounds [40].

## 4. Conclusions

In this paper, AlFe-PILC supported CrCe mixed oxides are synthesized and used for adsorption/desorption and catalytic combustion of CB. A series of characterization methods were used to investigate the structure and redox properties of these materials, including HRTEM-EDS, H_2_-TPR, TPD/TPSR, and O_2_-TPD. Comparing the results of the *S*_BET_, *V*_p_, and *d*_001_-value, AlFe-PILC performed better than Na-Mt. Without doubt, AlFe-PILC synthesized in this study constituted a class of porous materials with excellent properties. A large number of micro-mesoporosity and Ce was added to the AlFe-PILC to optimize its structure and improve the dispersion of Cr_2_O_3_ particles on the AlFe-PILC. XRD analysis and HRTEM images clearly revealed the stable layered structure with the *d*_001_ value ≈1.91 nm and well-dispersed active species in AlFe-PILC. The addition of Ce and optimized structure of support greatly improved the oxidative property of Cr_2_O_3_. CB-TPD experiments reveal that the optimized structure coupled with the strong interaction between CrCe metal oxides and AlFe-PILC enhanced CB adsorption capacity and adsorption strength. CB-TPSR results showed that the larger the overlapped region between CB desorption and the catalytic combustion, the better the catalytic performance. In particular, CrCe(5:1)/AlFe-PILC show an excellent catalytic property, and stability was due to the lower temperature of completely degraded CB (approximately 290 °C) and the conversion remained stable for 1000 h. CB catalytic combustion on CrCe/AlFe-PILC catalyst was via a Langmuir–Hinshelwood mechanism, and adjusting adsorption/desorption properties was one of the most important factors for designing efficient catalysts. CrCe/AlFe-PILC also exhibited good durability for CB destruction, both in the humid condition and in the presence of toluene; therefore, this catalyst deserves wide attention and it is a potential prospect for industrial application.

## Figures and Tables

**Table 1:** Characteristics of the samples: values of surface area and pore volume.

Samples	S_BET_ (m^2^/g)	*A*_mes_^a^ (m^2^/g)	*V*_p_^b^ (cm^3^/g)	*V*_mic_^c^ (cm^3^/g)
Na-Mt	51	41	0.076	0.0043
CrCe(5:1)/Na-Mt	22	22	0.058	-
AlFe-PILC	318	168	0.195	0.077
Cr/AlFe-PILC	221	71	0.165	0.066
CrCe(5:1)/AlFe-PILC	13	82	0.156	0.026

^a^ Calculated from BJH method. ^b^ Total pore volume estimated at P/P_0_ (relative pressure) = 0.99. ^c^ Calculated from the *t*-plot method.
